# Supplementary material for: Direct observation of intrinsic room-temperature ferroelectricity in 2D layered CuCrP2S6
Source: Nat Commun. 2023 Nov 11;14:7304. doi: 10.1038/s41467-023-43097-2 (PMC10640637; doi:10.1038/s41467-023-43097-2)
Supplement: Supplementary file 1 — Supplementary Information [file 41467_2023_43097_MOESM1_ESM.pdf]

SUPPLEMENTARY INFORMATION

Direct Observation of Intrinsic Room-temperature Ferroelectricity in 2D Layered $CuCrP_2S_6$

Weng Fu Io[1], Sin -Yi Pang[1], Lok Wing Wong[1], Yuqian Zhao[1], Ran Ding[1], Jianfeng Mao[1,2], Yifei Zhao[1], Feng Guo[1,2], Shuoguo Yuan[1], Jiong Zhao[1], Jiabao Yi[4], Jianhua Hao[1,2,3]*

[1] *Department of Applied Physics, The Hong Kong Polytechnic University, Hong Kong, P. R. China.*

[2]*The Hong Kong Polytechnic University Shenzhen Research Institute, Shenzhen 518057, P. R. China*

[3]*Photonics Research Institute, The Hong Kong Polytechnic University, Hong Kong, P.R. China*

[4] *Global Innovative Centre for Advanced Nanomaterials, College of Engineering, Science and Environment, The University of Newcastle, Callaghan, NSW 2308, Australia*

*Corresponding author.

E-mail address: jh.hao@polyu.edu.hk

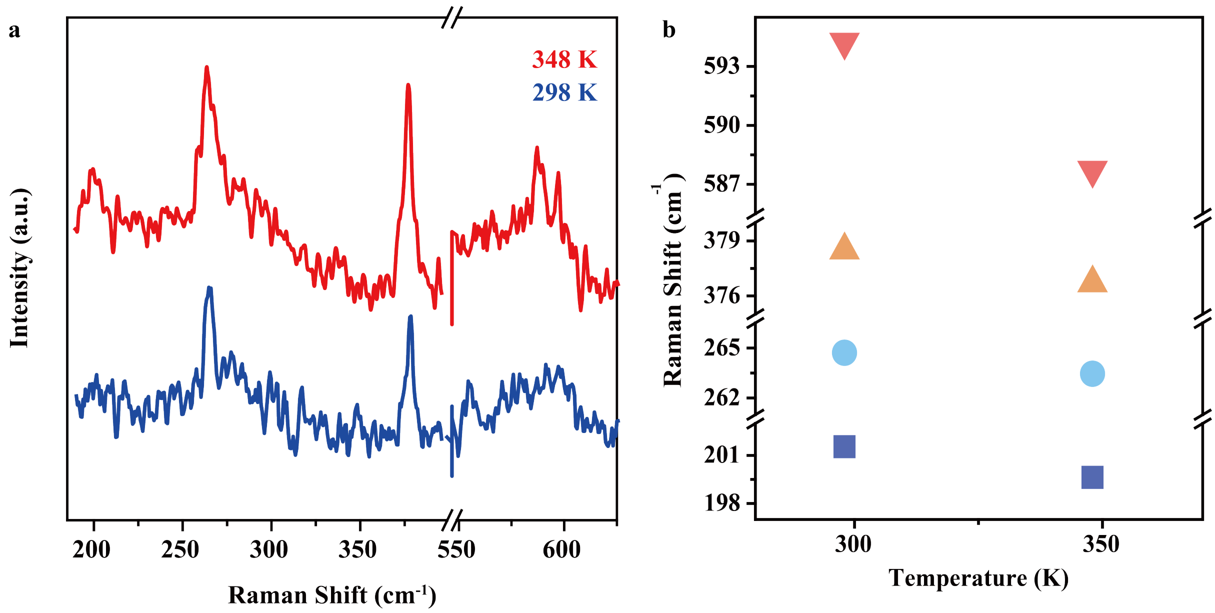

Figure S1. Raman characterization of CCPS samples. a Raman spectra of CCPS samples at 298 K (blue) and 348 K (red), respectively. **b** Temperature-dependent peak frequencies of the four vibrational modes in CCPS.

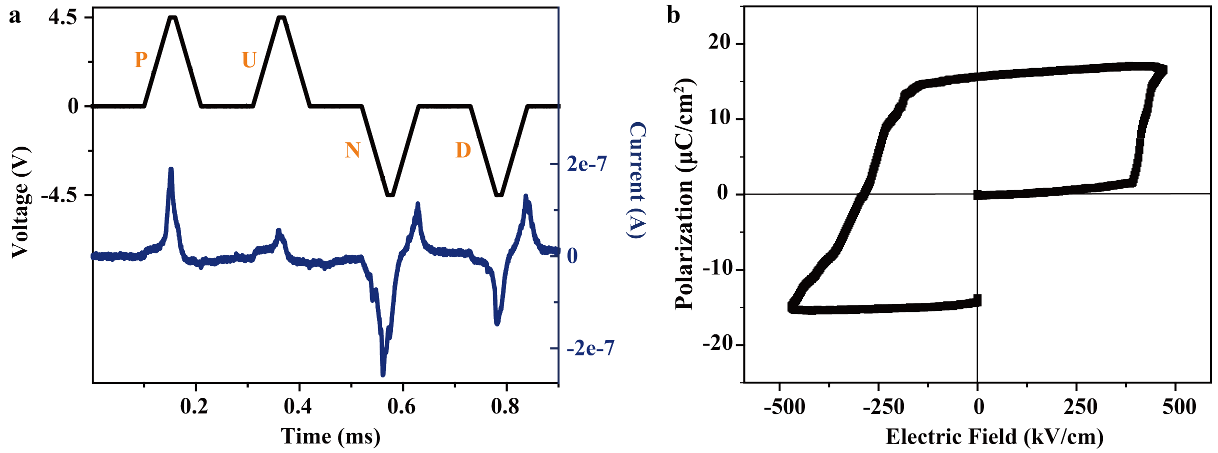

Figure S2. *P-E* hysteresis test of CCPS. a The voltage pulse (black) and current output (blue) in PUND measurement. **b** Calculated *P-E* hysteresis loop of CCPS with the thickness of 0.2 μm.

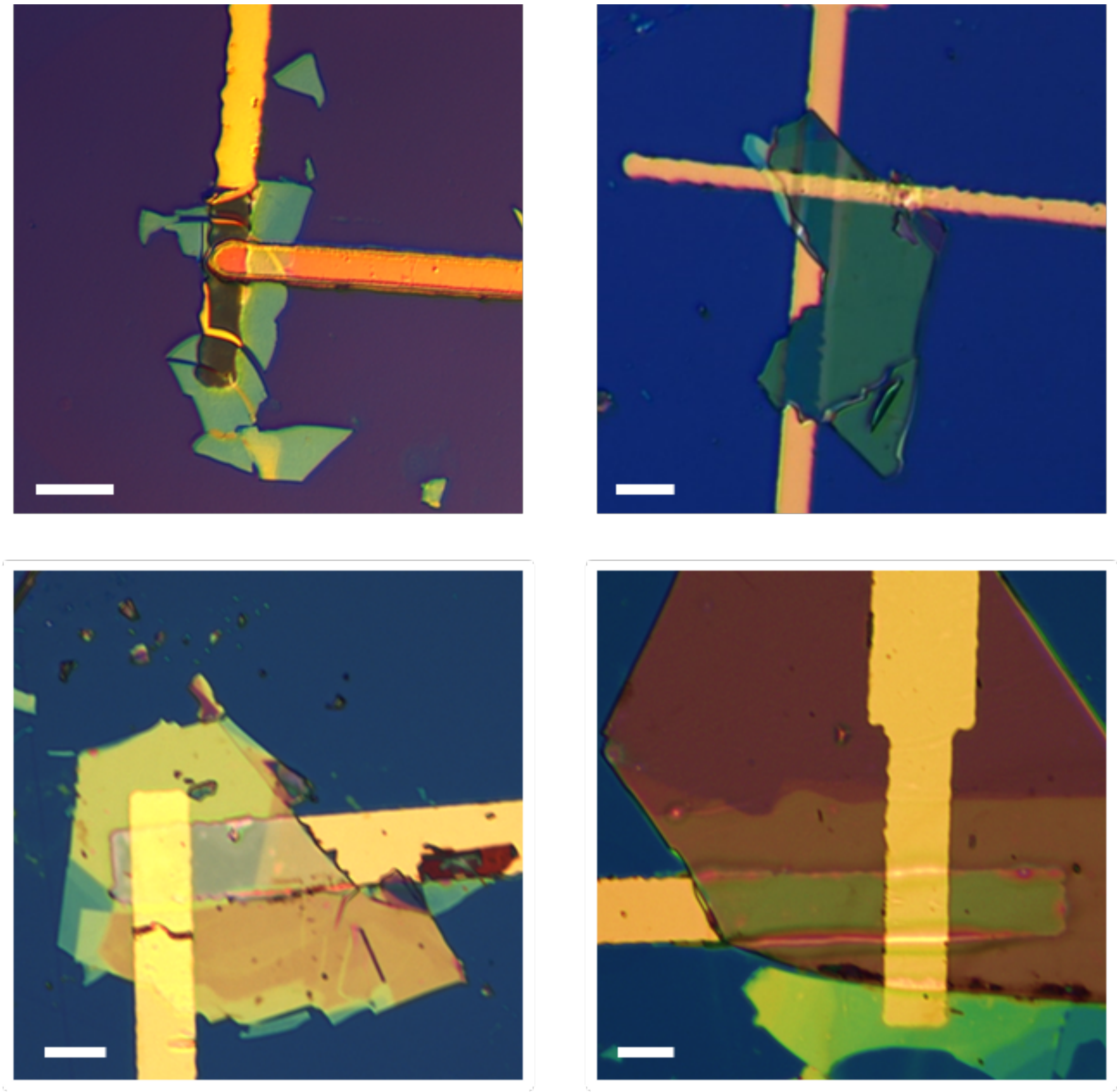

Figure S3. Optical images of some two-terminal Au/CCPS/Au vertical junction devices fabricated for *P-E* hysteresis measurements. Scale: 10 μm.

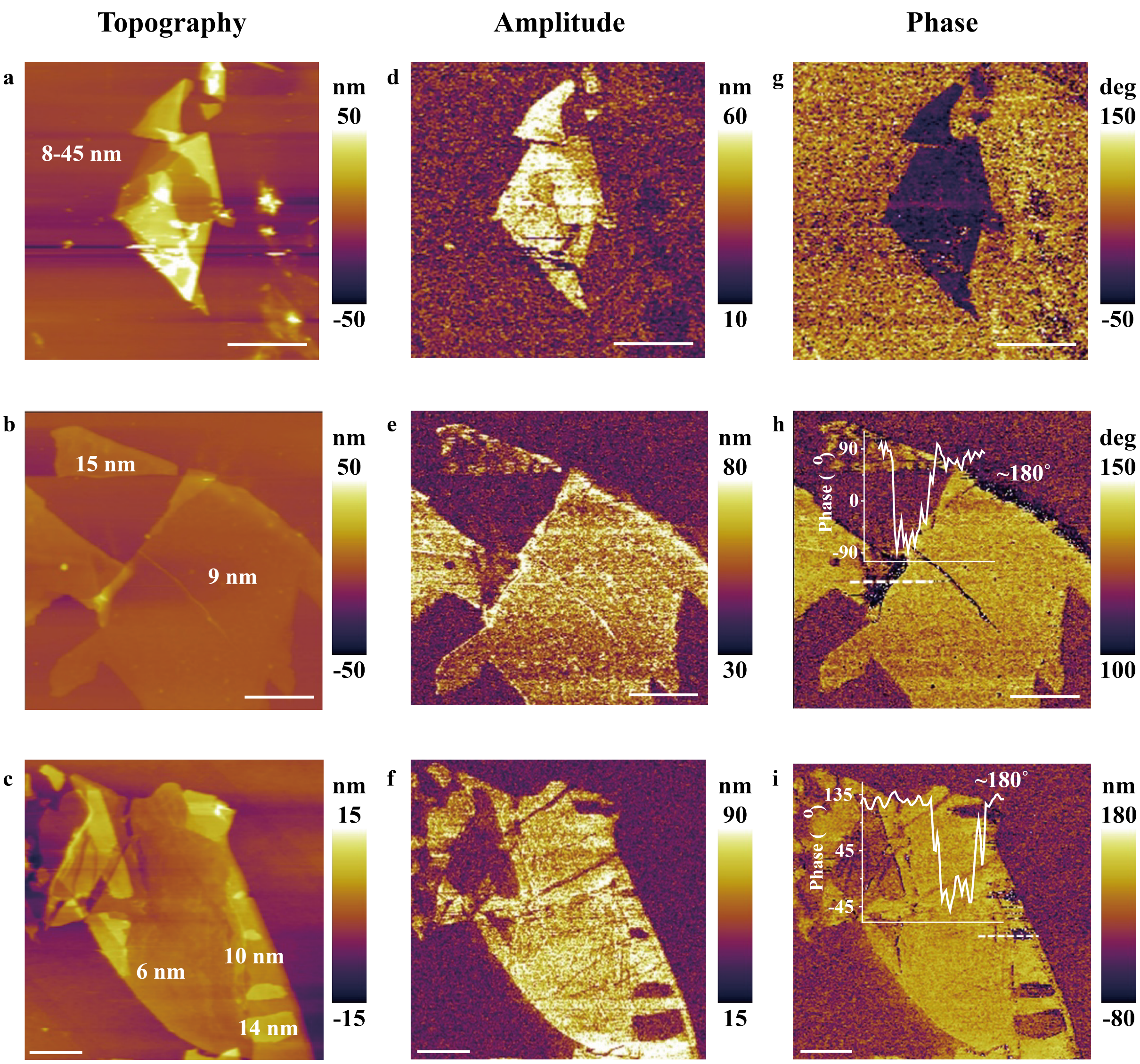

Figure S4. PFM characterization of CCPS samples. a-c AFM topographic images of CCPS nanoflakes and corresponding **d-f** amplitude and **g-i** phase images showing ferroelectric domains as visualized using vertical PFM mode. Scale: 10 μm, 2 μm, and 1 μm, respectively.

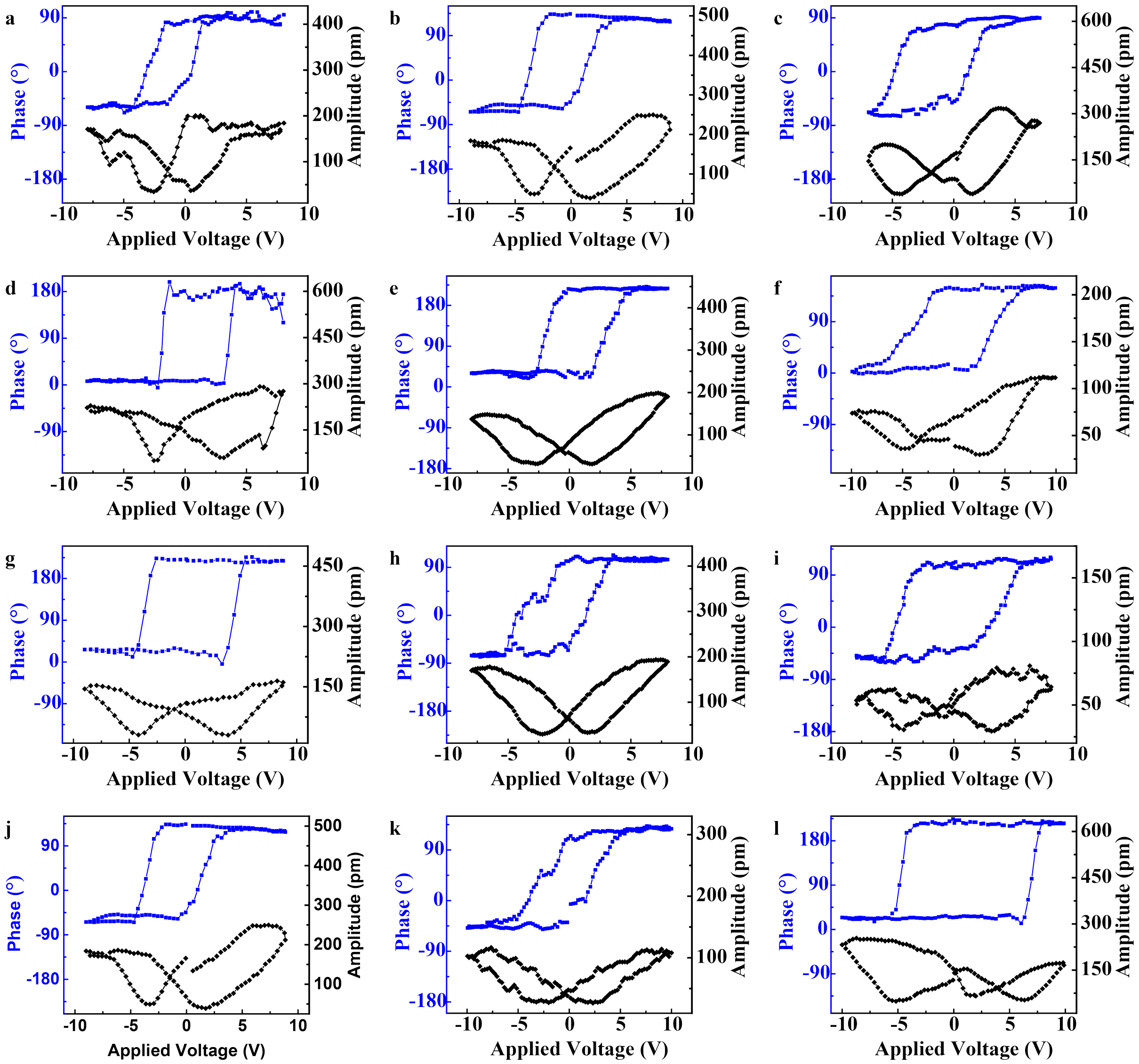

Figure S5. PFM hysteresis loop measurements of CCPS samples. a-l OOP off-field PFM phase (blue) and amplitude (black) hysteresis loops of CCPS samples with the thickness of 3.3, 4, 5, 6, 8, 17, 20, 40, 70, 79, 171, and 286 nm, respectively.

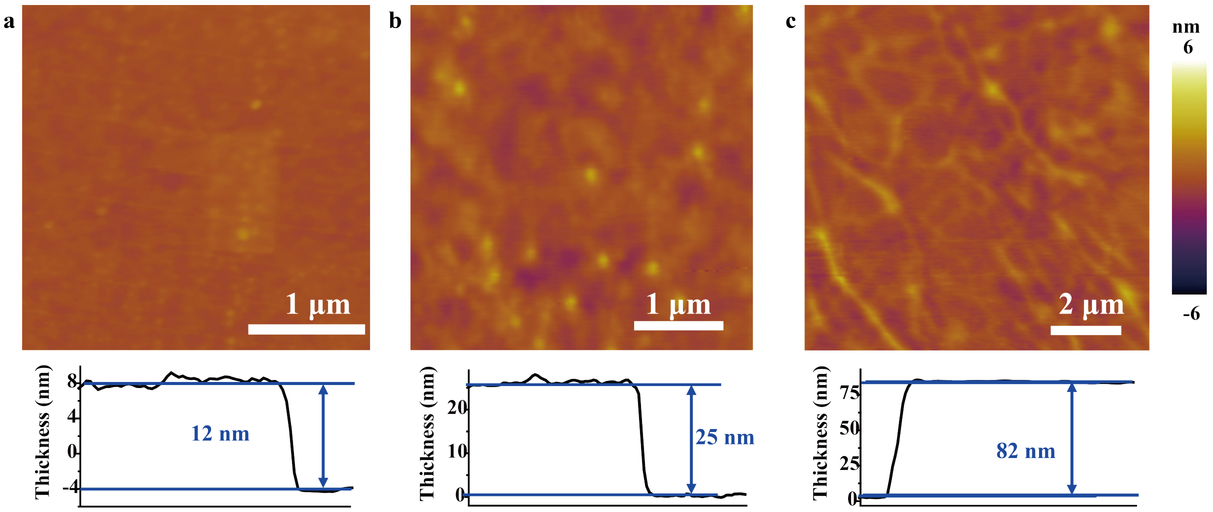

Figure S6. AFM characterization of CCPS samples. a-c AFM topographic images and height profiles of the CCPS nanoflakes corresponding to Figures 3a-c of the main text after applying the switching bias to modify the ferroelectric domain pattern.

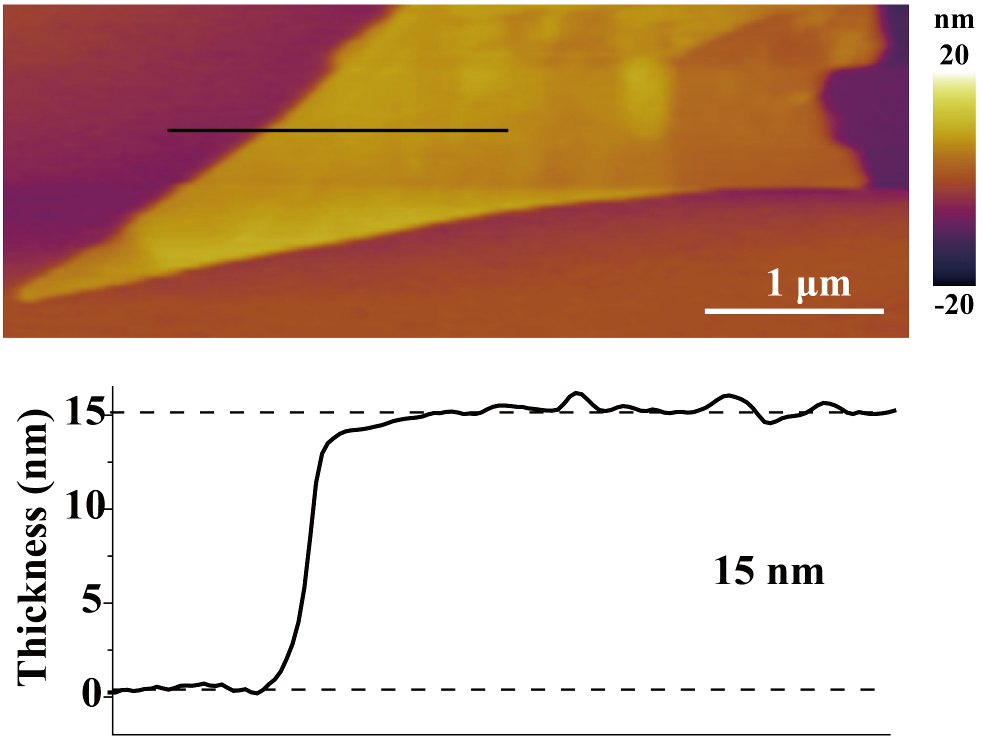

Figure S7. AFM topographic image and height profile of the CCPS sample in Figure 4 for temperature-dependent domain measurements.

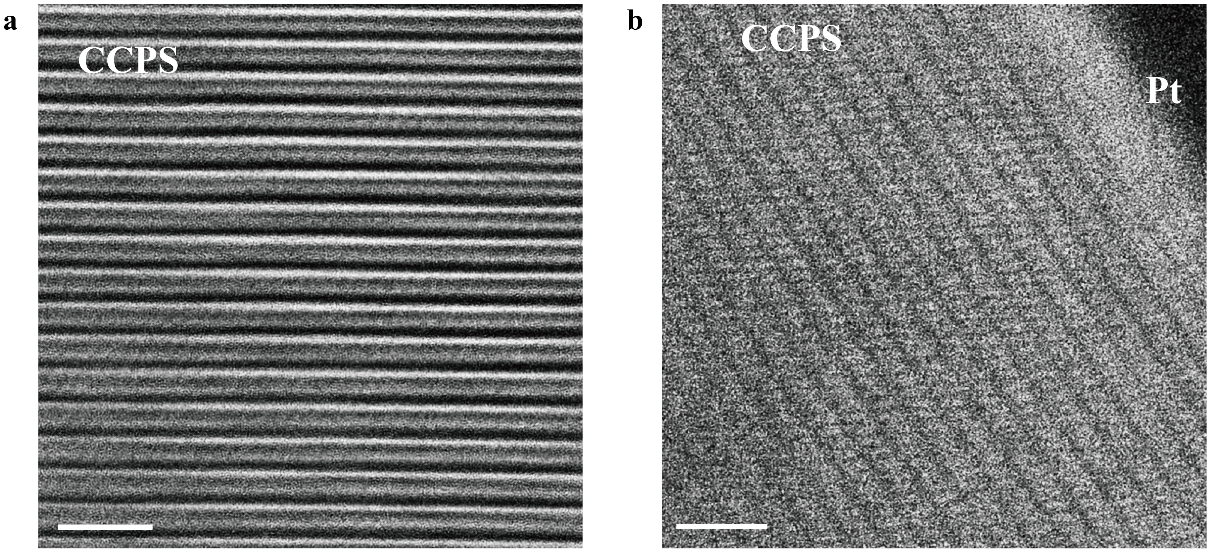

Figure S8. HAADF-STEM images of CCPS samples. a-b Corresponding HAADF-STEM images of the colored DPC-STEM profiles in Figure 5f and 5g, respectively. Scale: 2 nm.

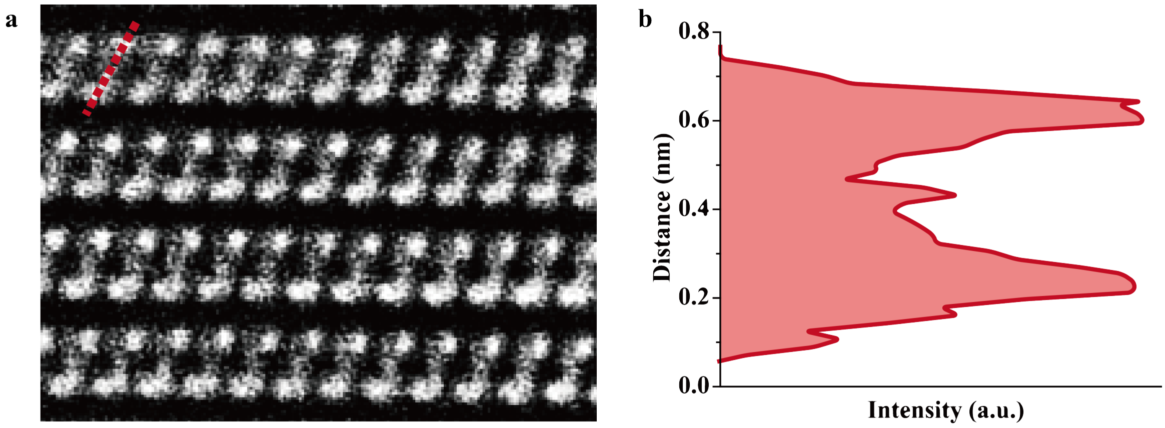

Figure S9. Atomic resolution HAADF-STEM analysis of CCPS. a HAADF-STEM image of CCPS at the paraelectric phase and **b** the corresponding intensity profile along the red dashed line.

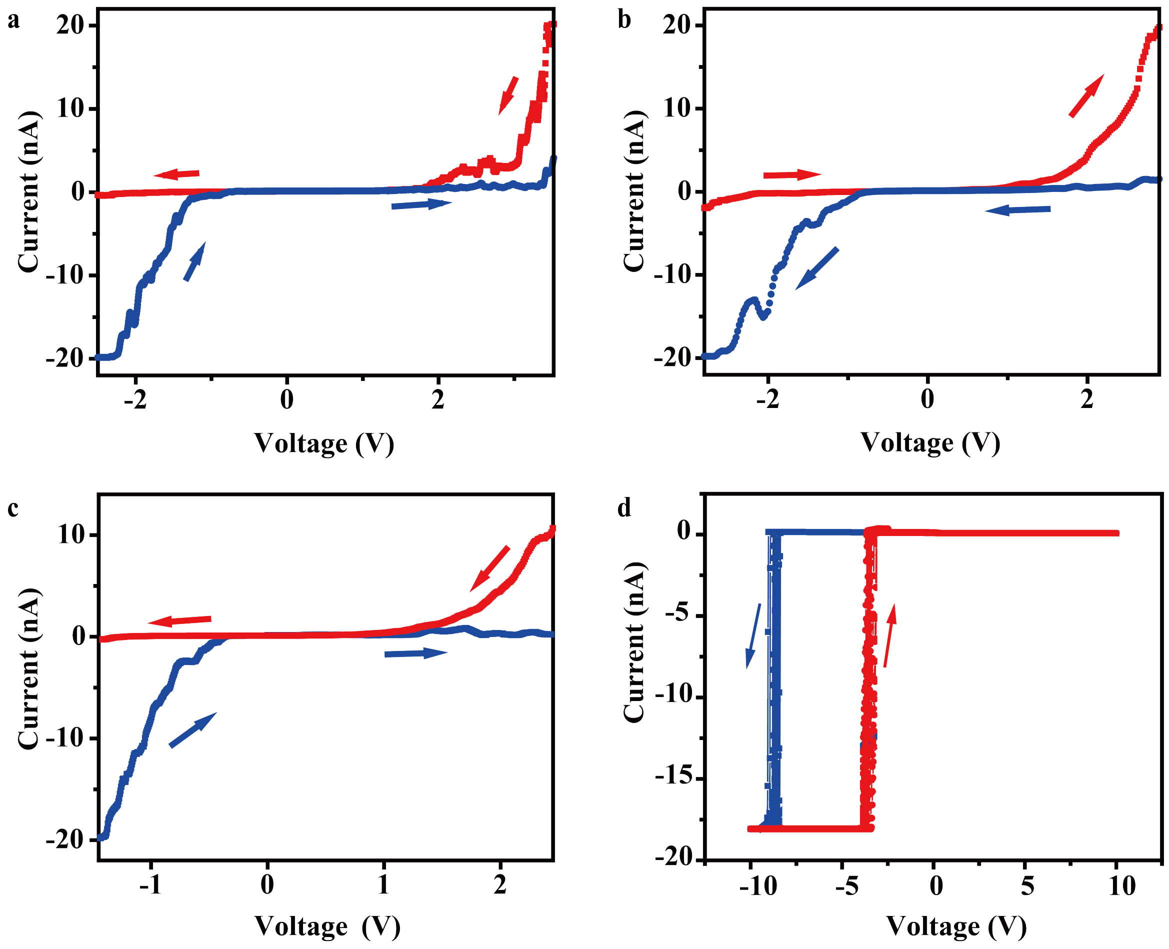

Figure S10. Electric characterization of CCPS-based ferroelectric diodes. a-c *I-V* characteristics of Pt/CCPS/Pt-based ferroelectric diodes with CCPS nanoflakes of ~ 7.2, 9.3, and 9.9 nm, respectively. **d** *I-V* characteristics of an Pt/CCPS/Graphene ferroelectric diode with a 20-nm-thick CCPS over 20 cycles.

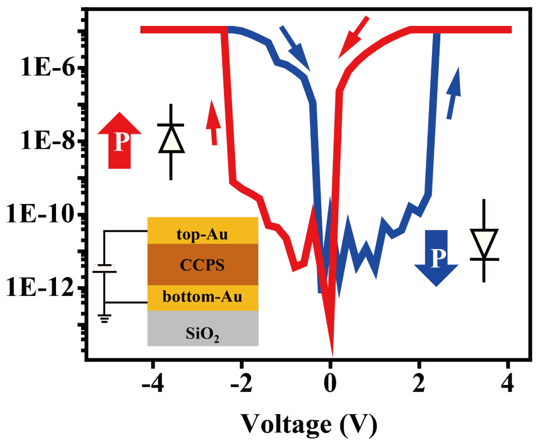

Figure S11. *I-V* characteristics of an Au/CCPS/Au diode with a thin ferroelectric CCPS film of thickness about 280 nm.

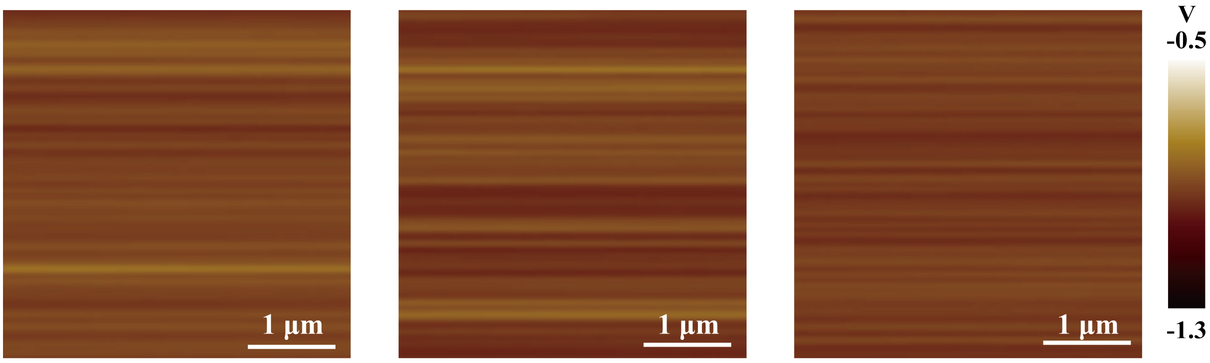

Figure S12. Calibration of the work function of conductive tip using highly oriented pyrolytic graphite (HOPG). Surface potential mappings of freshly cleaved HOPG at randomly selected regions.

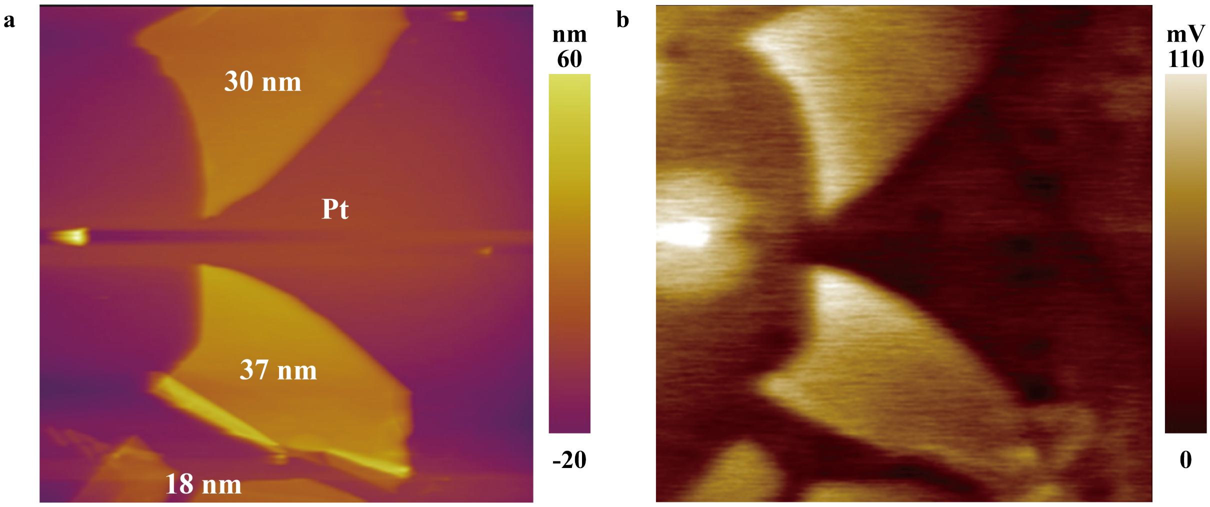

Figure S13. KPFM characterization of CCPS samples. a Surface topography and **b** surface potential images of exfoliated CCPS nanoflakes on Pt substrate.

From the SKPM mapping results in **Figure S12**, the contact potential differences (V_{CPD}) between the Pt/Ir-coated tip and HOPG are -0.868 V, -0.847 V and -0.870 V, respectively, and the calculated average contact potential difference (V_{CPD}) is about -0.862 V. The work function of freshly cleaved HOPG has a work function of ~4.6 eV. The work function of the Pt/Ir-coated tip $\phi_{Pt/Ir} = \phi_{HOPG} - eV_{CPD}(HOPG) = 5.462$ eV. As shown in **Figure S13**, the V_{CPD} between the Pt/Ir-coated tip and CCPS nanoflakes is about 75 mV. The work function of the CCPS nanoflakes ϕ_{CCPS} can be calculated by $\phi_{CCPS} = \phi_{Pt/Ir} + eV_{CPD}(CCPS) = 5.54$ eV.

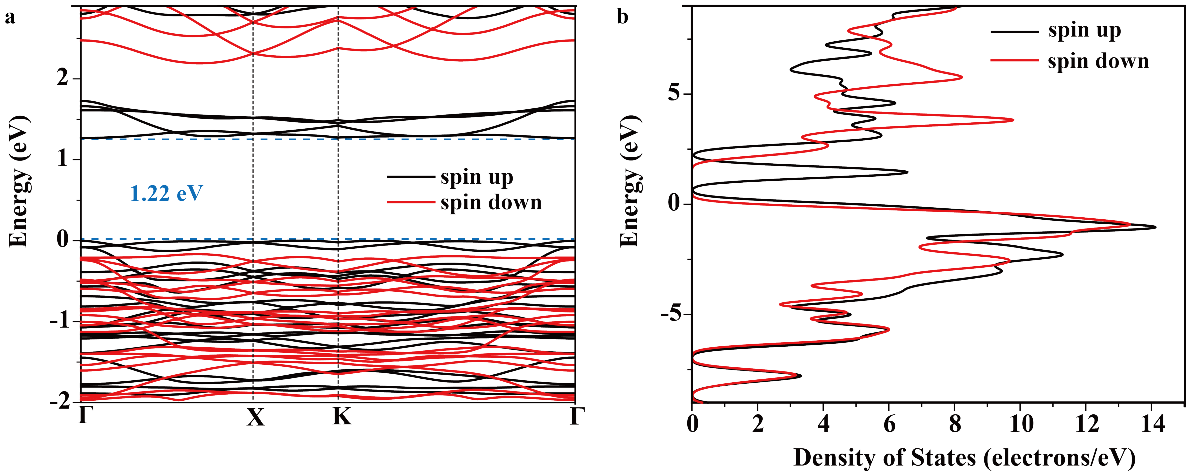

Figure S14. Simulation results of electronic band structure. a Band structure and **b** density of states of ferroelectric phase CCPS.

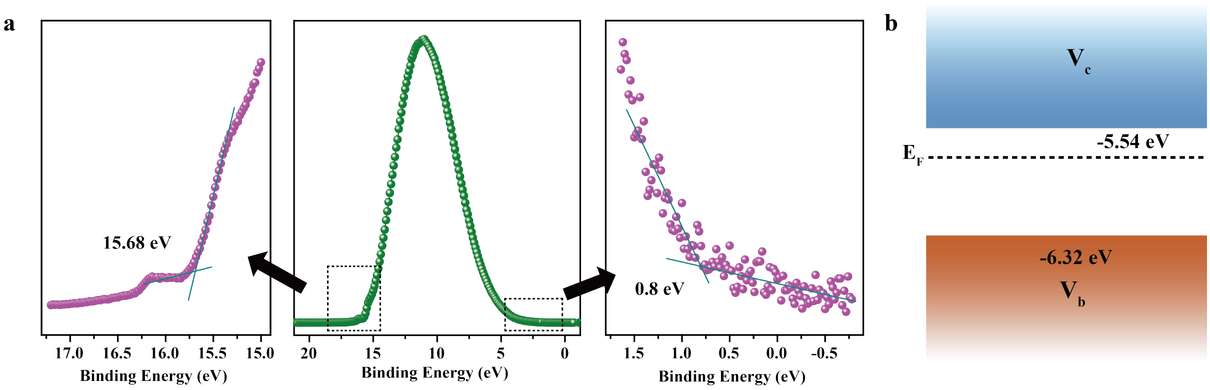

Figure S15. Energy band diagram of CCPS. a UPS analysis of the vdW layered CCPS. **b** Sketched energy band diagram of CCPS based on the UPS and SKPM measurement results.

Selection of the atomic structure of CCPS at the paraelectric phase for DFT simulation

Regarding the partial Cu occupations in two off-center positions and the overall zero net polarization in the paraelectric state, as verified by our experimental STEM observation and previous literature, we first determined the possible atomic configurations by specifying the Cu coordinates and 12 different configurations were considered in a 2 × 2 × 1 supercell. All of the simulated CCPS structures with different Cu ion orderings show very similar total energies (E_i) of around -390 eV, evidencing the disordered partial Cu occupations. Despite the small difference, the CCPS structure with alternating off-centered Cu locations exhibits the lowest E_i of -391.67 eV and is thus selected as the representative paraelectric structure for charge density difference calculation as presented in Figure 5h.
